# The Connecticut Veterinary Medical Association

**Published:** 1892-02

**Authors:** Rob’t S. Todd

**Affiliations:** Secretary


*The Connecticut Veterinary Association.*—A regular meeting of the above Association was held at Dr. Thomas Bland's new Veterinary Infirmary, Waterbury, on Tuesday, December 1st, 1891. The President, Dr. Thos. Bland, occupied the chair. The members present were Drs. Ross, Bates, Beckley, Towne, Heath, E. A. McLellan and Todd. Dr. T. J. Sabin, of Lynn, Mass., was also present. As a number of the members had to leave for home on early trains the meeting was not a lengthy one.

Resolutions were adopted upon the death of Ex-President Dr. Geo. Bridges. The President spoke upon the subject of Pre Veterinary Education and hoped the colleges would soon refuse to admit students whose limited education was a bar to the advancement of our profession, and said that as long as the colleges continued to admit men whose education was barely on a par with the common hostler we need not expect to be recognized as anything but vulgar *horse doctors*, and *how can* we expect to receive much from our State Legislature, etc. A short discussion ensued. It was decided to hold the next meeting at New Haven on the first Tuesday in March.

ROB'T S. TODD, *Secretary.*

